# The Effects of Simvastatin on Proteinuria and Renal Function in Patients with Chronic Kidney Disease

**DOI:** 10.1155/2015/485839

**Published:** 2015-10-12

**Authors:** Bancha Satirapoj, Anan Promrattanakun, Ouppatham Supasyndh, Panbuppa Choovichian

**Affiliations:** Division of Nephrology, Department of Medicine, Phramongkutklao Hospital and College of Medicine, Bangkok 10400, Thailand

## Abstract

Current data suggests that statins might have beneficial effects on renal outcomes. Beneficial effects of statin treatment on renal progression in advanced chronic kidney disease (CKD) are obviously controversial. In a retrospective, controlled study, the authors have evaluated the effects of 53-week treatment with simvastatin, versus no treatment on proteinuria and renal function among 51 patients with CKD stages III-IV. By the end of the 53-week treatment, urine protein excretion decreased from 0.96 (IQR 0.54, 2.9) to 0.48 (IQR 0.18, 0.79) g/g creatinine (*P* < 0.001) in patients treated with simvastatin in addition to ACEI and ARBs, while no change was observed among the untreated patients. Moreover, a significantly greater decrease in urine protein excretion was observed in the simvastatin group as compared with the untreated group. The mean changes of serum creatinine and eGFR did not significantly differ in both groups. A significantly greater decrease in total cholesterol and LDL-cholesterol was found in the simvastatin group than in the untreated group. In summary, apart from lipid lowering among CKD patients, ingesting simvastatin was associated with a decrease in proteinuria. These statin effects may become important for supportive therapy in renal damage in the future.

## 1. Introduction

Chronic kidney disease (CKD) is a common condition and its prevalence is increasing worldwide [1]. According to data from the health information on subjects at the Armed Forces Research Institute of Medical Sciences, Thailand, the overall prevalence of patients with CKD is 7.5% [2]. The importance of understanding modifiable risk factors serves as a basis for devising treatment strategies to prevent the development and progression of CKD [3]. CKD patients frequently manifest dyslipidemia, such as hypercholesterolemia, as well as hypertension [4]. Extensive knowledge about abnormal lipid patterns among patients with advanced CKD and elevated total cholesterol, high non-HDL cholesterol, a high ratio of total cholesterol/HDL, and low HDL in particular was significantly associated with an increased risk of developing renal dysfunction [5]. Hyperlipidemia has been hypothesized to play an important role in the progression of renal injury [6].

Use of statins is beneficial for most patients with CKD who are at high cardiovascular risk [7], although research is needed to ascertain how to best prevent kidney injury. Experimental evidence suggests that statin can prevent the progression of kidney injury [3]. However, studies among humans on the subject are scarce. In meta-analysis, claims of improved renal outcomes have been made, encouraging broader adoption of statins among patients with predialysis CKD [8, 9]. Renoprotective effects of statins remain uncertain because of relatively sparse data and possible outcome reporting bias [10]. The aim of our study was to evaluate the efficacy and safety of statins for renal outcomes in advanced stages of proteinuric CKD.

## 2. Materials and Methods

### 2.1. Subjects and Study Design

The research employed a retrospective cohort-based design that randomly used medical records from April 2012 to March 2013 on CKD patients who routinely visited an outpatient facility including stable blood pressure and blood glucose within 3 months. Inclusion criteria of the study included age, 18 years or older, proteinuric CKD > 300 mg/day, and urinary protein creatinine ratio (UPCR) test before and after initiating simvastatin for 53 weeks. Treatment group of patients received simvastatin 10–40 mg daily. The other patients in the normal care group were treated according to their physician's standard of care. Normal care included life style changes, such as low fat diet, weight loss, and exercise, in addition to all necessary drug treatment without statins. Exclusion criteria included active malignancy, severe heart, lung, or liver disease, stroke, chronic infection, for example, tuberculosis, within one year of starting the study, and any immunological or inflammatory disorders.

### 2.2. Data Collection

From their clinical data, we determined the effects of statins on renal parameters after a 53-week period. At the time of entry, all patients were also taking standard antihypertensive agents including angiotensin receptor blockers, angiotensin converting enzyme inhibitors, calcium channel blocker, beta-blockers, alpha-blockers, and diuretics. A complete medical history was taken and physical examination was performed on all subjects. All subjects fasted for at least 12 hours overnight before all blood drawing. Complete blood counts, blood urea nitrogen, serum creatinine, and comprehensive serum chemistries were measured. The serum concentration of creatinine using the enzymatic method was determined with reagents from Roche Diagnostics (Mannheim, Germany) and the calibrator was IDMS standardized. Glomerular filtration rate (GFR) was estimated from calibrated serum creatinine with the 2009 CKD-EPI creatinine equation [11]. Random urine samples were collected from patients. Urinary protein and creatinine concentrations were measured and expressed as the UPCR. The study was approved by the Institutional Review Board of the Royal Thai Army Medical Department, Bangkok, Thailand. All participants gave their written informed consent.

### 2.3. Statistical Analysis

Results are expressed as the mean ± SD, as medians with interquartile ranges, or as a percentage in categorical variables. Differences between groups mean or median values were evaluated using the independent Student's *t*-test or Mann-Whitney test. In the case of continuous variables measured at the baseline and the end of study, differences within the group were analyzed by paired *t*-test or Wilcoxon Signed ranks test. Statistical significance was defined as *P* < 0.05. All analyses were performed with SPSS Software (SPSS, Inc., Chicago, IL, USA, Version 17).

## 3. Results

### 3.1. Patient Characteristics

Patient characteristics are shown in Table 1. Blood pressure was fairly well controlled and most patients were taking renin angiotensin system (RAS) inhibitors (68-69%). Diuretics were prescribed for 14 patients, alpha blockers were prescribed for 14 patients, and calcium channel blockers were prescribed for 12 patients. The mean simvastatin dose was 28 mg/day. Average estimated GFR was 40.10 ± 26.55 mL/min/1.73 m^2^ and average UPCR was 1.92 ± 1.95 g/g creatinine. Kidney diseases among 51 of the study patients comprised glomerular diseases (*N* = 20) and chronic tubulointerstitium disease (*N* = 1) and the remaining 30 patients were not well characterized. Underlying disease of type 2 diabetes was significantly higher among patients treated with simvastatin (68%) than in patients with nonsimvastatin group (30.8%). Age, sex, body weight, systolic blood pressure, diastolic blood pressure, BUN, serum creatinine, estimated GFR, UACR, cholesterol, low density lipoprotein (LDL) cholesterol, high density lipoprotein (HDL) cholesterol, and triglycerides did not differ at baseline between the simvastatin and nonsimvastatin groups (Table 1).

### 3.2. Effect of Simvastatin Use on Metabolic Profiles

Both systolic and diastolic blood pressure remain unaltered during the observation period. However, lipid profiles improved after 53 weeks of simvastatin treatment. Mean changes of total cholesterol (−52.44 ± 106.05 versus −6.65 ± 36.88 mg/dL, *P* = 0.045), (LDL-C −41.28 ± 79.96 versus −3.81 ± 25.44 mg/dL, *P* = 0.033), and non-HDL-C (−56.04 ± 111.34 versus −8.50 ± 29.78 mg/dL, *P* = 0.049) were significantly reduced in the simvastatin group when compared with the nonsimvastatin group (Table 2).

### 3.3. Effect of Simvastatin Use on Renal Outcomes

Renal function did not significantly change in both groups. Finally, mean changes of serum creatinine (0.1 ± 0.62 versus 0.1 ± 0.68 mg/dL, *P* = 0.308) and mean changes of estimated GFR (−1.2 ± 7.2 versus −3.3 ± 11.9 mL/min/1.73 m^2^, *P* = 0.458) did not significantly differ in the simvastatin and nonsimvastatin groups.

Individual CKD patients with proteinuria >300 mg/day receiving simvastatin had a statistically significant decreased UPCR from 0.96 (IQR 0.54, 2.9) to 0.48 (IQR 0.18, 0.79) g/g creatinine (*P* < 0.001) at 53 weeks after treatment, but no significant change was observed in the nonsimvastatin group (1.41 (IQR 0.66, 2.41) to 1.21 (IQR 0.19, 1.56) g/g creatinine). Moreover, mean change of UPCR also significantly differed in the simvastatin and nonsimvastatin groups (Table 3).

### 3.4. Safety

No unexpected safety concerns were identified and similar incidences of adverse events were experienced in each of the treatment groups. No serious adverse effects such as persistent elevations in liver function enzymes and creatine phosphokinase values were observed in those using simvastatin.

## 4. Discussion 

The present study reported that simvastatin was associated with lipid lowering and antiproteinuric benefits in patients with moderate to advanced CKD. They seemed to be safe with CKD, with respect to the risk of hepatotoxicity. However, the present study could not clearly confirm evidence of any renoprotective effect of statins in patients with CKD, as indicated by no difference found in GFR and serum creatinine between simvastatin and control groups.

Patients with higher levels of proteinuria have an increased risk of severe CKD and as a predictor of future decline in GFR [12], but limited therapeutic options are available to decrease proteinuria. In the PLANET I study among patients with diabetic nephropathy and the PLANET II study among patients without diabetic nephropathy, atorvastatin significantly reduced proteinuria by 15% to 23.8%. Recent meta-analyses of randomized, placebo-controlled trials among patients have suggested that statins were associated with reductions in high levels of proteinuria [9]. As was consistent with our study, statins produced a beneficial effect on pathologic proteinuria in CKD populations. However, the reduction of proteinuria in our study might be the effect of decreasing renal function in the simvastatin treated group.

The beneficial effect of statins on proteinuria seen in our study may be potentially explained by cholesterol dependent effects and cholesterol independent effects. Experimental studies have documented that dyslipidemia contributes to glomerular and interstitial injury and the severity of the hypercholesterolemia correlates with proteinuria [13]. In addition to the beneficial effects of lowering lipids, statins also influence important intracellular pathways that are involved in the inflammatory and fibrogenic responses, the main pathway of progressive renal injury [14, 15]. Moreover, the reasons to favor the use of statins in CKD include beneficial effects on endothelial function, suppressing monocyte recruitment, mesangial cell proliferation and mesangial matrix accumulation, antifibrotic effects, and antioxidation and anti-inflammatory cytokines [13, 16].

Our findings are consistent with a recent study, reporting that urinary protein losses had fallen, but renal function was stable among CKD patients at the end of one year of therapy with intensified lipid-lowering statin [17]. Intensified lipid-lowering therapy did not appear to have any GFR effect. Another meta-analysis also showed that statins significantly reduced total cholesterol but did not improve GFR [10]. However, data from several small studies and subgroup analysis from main studies suggest that statins might slow CKD progression. The secondary coronary heart disease prevention GREACE study suggested that dose titration with atorvastatin prevented creatinine clearance decline and significantly improved renal function among patients with coronary heart disease and normal GFR [18]. A post hoc analysis of the Cholesterol and Recurrent Events (CARE) trial reported slow renal function loss with the use of pravastatin in patients with previous myocardial infarction and moderate to severe CKD, especially among those with proteinuria [19]. The published heart protection study (HPS) subgroup analysis for participants with diabetes mellitus also showed that simvastatin significantly decreased the rise in serum creatinine in patients with and without diabetes mellitus [20]. All previous subgroup analyses suggested that statin was associated with a significantly smaller fall in the estimated GFR compared with the placebo group. In addition, as a post hoc analysis, using estimates of renal function, some limitations were observed in interpreting these data, so a small proportion of patients, who had advanced CKD, were included in this analysis, whereas our findings in the simvastatin group revealed estimated GFR did not improve, but no significant decline was observed among advanced CKD subjects. Therefore, the available data on statin with GFR in CKD patients are still conflicting, because of possible outcome reporting bias.

One possible explanation of estimated GFR improvement in previous studies may be related to the intensity of statin therapy. Improvement in estimated GFR occurred with low-dosage atorvastatin (10 mg/day), but high-dosage atorvastatin (80 mg/day) demonstrated significantly greater improvement in estimated GFR than that achieved by low-dosage atorvastatin [21]. Previous studies with moderate to high doses of statins demonstrated a slowing in renal function decline [17, 19, 20]. Mild to moderate doses of simvastatin were used in our study, and, therefore, they might have produced a negative GFR effect of simvastatin in proteinuric CKD patients.

Our study had limitations that should be considered. First, this was a retrospective controlled study, and the limitations of it are well described. Thus, other confounding factors and change of metabolic parameters might have affected the proteinuria in the simvastatin group during the follow-up period. Although a significant reduction of urinary protein excretion in the statin treated group and mean changes of urine protein between the statin treated and nonstatin treated groups were observed, differences in baseline data of comorbid diseases included type 2 diabetes, hypertension, and medications between both groups. Second, renal outcomes in the current study were estimated using the CKD-EPI-GFR formula and UPCR, which are less accurate than nuclear isotope estimates of GFR and 24-hour urine protein. Finally, this study enrolled a small sample size and had a short duration of follow-up. However, our study revealed that individuals with moderate to severe kidney disease may derive clinically relevant proteinuric benefits from the use of simvastatin, especially those with proteinuria. These findings should be confirmed by a large randomized trial conducted specifically among this patient population.

## 5. Conclusion

This study has showed that treatment with statins in addition to a regimen with ACE inhibitors or ARBs can reduce proteinuria in patients with proteinuric CKD and hyperlipidemia. The benefits appear to occur in addition to those treated with standard CKD management.

## Figures and Tables

**Table 1:** Patients' characteristics.

	Simvastatin (*N* = 25)	Nonsimvastatin (*N* = 26)	*P* value

Male (%)	17 (68%)	20 (76.9%)	0.556
Age (year)	62 ± 19.86	60.04 ± 21.31	0.735
Weight (kg)	67.80 ± 13.92	63.34 ± 13.80	0.256
Primary renal diseases, *N* (%)			0.612
Glomerular diseases	11	9	
CTIN	—	1	
Unknown	14	16	
Diabetes mellitus (%)	17 (68%)	8 (30.8%)	0.012
Hypertension (%)	21 (84%)	17 (65.4%)	0.199
Atherosclerosis (%)	6 (24.0%)	5 (19.2%)	0.743
Current antihypertensive agents (%)			
ACEI/ARB	17 (68.0%)	18 (69.2%)	0.572
Diuretics	9 (36.0%)	5 (19.2%)	0.220
Alpha-blockers	9 (36.0%)	5 (19.2%)	0.220
Beta-blockers	5 (20.0%)	3 (11.5%)	0.171
CCB	7 (28.0%)	5 (19.2%)	0.258
SBP (mmHg)	139.08 ± 17.24	137.38 ± 17.88	0.732
DBP (mmHg)	79.44 ± 14.22	77.27 ± 13.26	0.575
UPCR (g/g creatinine)	0.96 (0.54, 2.9)	1.41 (0.66, 2.41)	0.445
GFR (mL/min/1.73 m^2^)	41.12 ± 28.97	39.77 ± 23.55	0.856
BUN (mg/dL)	27.75 ± 12.68	30.60 ± 15.16	0.472
Serum creatinine (mg/dL)	1.97 ± 0.72	1.97 ± 0.73	0.995
Cholesterol (mg/dL)	211.72 ± 123.80	185.62 ± 57.15	0.335
Triglyceride (mg/dL)	158.48 ± 154.03	143.04 ± 112.93	0.684
HDL (mg/dL)	52.24 ± 15.05	57.19 ± 27.02	0.425
LDL (mg/dL)	134.04 ± 93.90	109.00 ± 42.80	0.232
Non-HDL (mg/dL)	157.56 ± 126.09	122.27 ± 55.55	0.199
AST (mg/dL)	24.80 ± 11.39	23.69 ± 10.88	0.724
ALT (mg/dL)	23.44 ± 19.07	21.04 ± 13.36	0.604

Values expressed as mean ± SD or median with interquartile ranges, ACEI: angiotensin converting enzyme inhibitor; ARB: angiotensin type 1 receptor blocker; ALT: alanine aminotransferase; AST: aspartate aminotransferase; BUN: blood urea nitrogen; CCB: calcium channel blockers; DBP: diastolic blood pressure; HDL: high density lipoprotein; LDL: low density lipoprotein; GFR: glomerular filtration rate; SBP: systolic blood pressure; UPCR: urine protein creatinine ratio.

**Table 2:** Changes of metabolic profiles after 53 weeks of statin treatment.

Variables	Mean changes		*P* value
	Simvastatin	Nonsimvastatin	
SBP (mmHg)	−5.72 ± 21.88	−0.77 ± 20.19	0.405
DBP (mmHg)	−1.84 ± 13.89	−4.50 ± 13.64	0.493
Cholesterol (mg/dL)	−52.44 ± 106.05	−6.65 ± 36.88	0.045
Triglyceride (mg/dL)	−34.68 ± 140.77	−15.81 ± 83.44	0.561
HDL (mg/dL)	0.36 ± 15.95	1.65 ± 20.31	0.802
LDL (mg/dL)	−41.28 ± 79.96	−3.81 ± 25.44	0.033
Non-HDL (mg/dL)	−56.04 ± 111.34	−8.50 ± 29.78	0.049
AST (U/L)	−2.12 ± 7.57	5.15 ± 18.83	0.079
ALT (U/L)	3.36 ± 28.03	10.96 ± 44.41	0.470

Data are expressed as mean changes of 53 weeks ± SD; ALT: alanine aminotransferase; AST: aspartate aminotransferase; DBP: diastolic blood pressure; HDL: high density lipoprotein; LDL: low density lipoprotein; SBP: systolic blood pressure.

**Table 3:** Renal outcomes after 53 weeks of statin treatment.

Variables	Simvastatin				Nonsimvastatin				*P* value between groups^(&)^
	(*N* = 25)				(*N* = 26)				
	Baseline	At 53 weeks	Mean differences	*P* value^(#)^	Baseline	At 53 weeks	Mean differences	*P* value^(#)^	
Mean UPCR (g/g creatinine)	2.26 ± 2.99	0.83 ± 0.95	−1.4 ± 2.8	0.019	1.79 ± 1.47	1.77 ± 2.68	−0.02 ± 2.4	0.969	0.049
Median UPCR (g/g creatinine)	0.96 (0.54, 2.9)	0.48 (0.18, 0.79)		<0.001	1.41 (0.66, 2.41)	1.21 (0.19, 1.56)		0.485	
GFR (mL/min/1.73 m^2^)	41.12 ± 28.97	39.88 ± 29.74	−1.2 ± 7.2	0.396	39.77 ± 23.55	36.46 ± 18.82	−3.3 ± 11.9	0.168	0.458
Serum creatinine (mg/dL)	1.97 ± 0.73	2.06 ± 0.72	0.1 ± 0.62	0.315	1.97 ± 0.72	2.07 ± 0.67	0.1 ± 0.68	0.458	0.308

Values expressed as mean ± SD or median with interquartile ranges; GFR: glomerular filtration rate; UPCR: urine protein creatinine ratio; (#) comparisons within groups; (&) comparisons between groups.
